# Challenges and Perspectives for Biosensing of Bioaerosol Containing Pathogenic Microorganisms

**DOI:** 10.3390/mi12070798

**Published:** 2021-07-05

**Authors:** Meixuan Li, Lei Wang, Wuzhen Qi, Yuanjie Liu, Jianhan Lin

**Affiliations:** Key Laboratory of Agricultural Information Acquisition Technology, Ministry of Agriculture and Rural Affairs, China Agricultural University, Beijing 100083, China; 2018308130117@cau.edu.cn (M.L.); wanglei123@cau.edu.cn (L.W.); wuzhen.qi@cau.edu.cn (W.Q.); yjliu@cau.edu.cn (Y.L.)

**Keywords:** bioaerosol, collection, biosensing, microfluidic chip, pathogenic microorganisms

## Abstract

As an important route for disease transmission, bioaerosols have received increasing attention. In the past decades, many efforts were made to facilitate the development of bioaerosol monitoring; however, there are still some important challenges in bioaerosol collection and detection. Thus, recent advances in bioaerosol collection (such as sedimentation, filtration, centrifugation, impaction, impingement, and microfluidics) and detection methods (such as culture, molecular biological assay, and immunological assay) were summarized in this review. Besides, the important challenges and perspectives for bioaerosol biosensing were also discussed.

## 1. Introduction

Bioaerosols within the diameter of 100 µm mainly refer to bacteria, viruses, fungi, and some microbial fragments suspended in the air [1,2,3]. The size of bioaerosols containing fungi, bacteria, and viruses generally range from 1 to 30 μm, from 0.25 to 8 μm and less than 0.3 μm, respectively [4,5]. According to the report from the World Health Organization, lower respiratory infections remained the world’s most deadly communicable disease and were ranked as the 4th leading cause of death, resulting in 2.6 million deaths in 2019 [6]. Severe Acute Respiratory Syndrome (SARS) in 2003 [7,8], H1N1 Influenza in 2009 [9,10], Middle East Respiratory Syndrome in 2013 [11,12,13], and Coronavirus (COVID-19) pandemic in 2019 [14,15,16] could also be spread through bioaerosols, which pose a great threat to global public health. Therefore, it is vital to monitor bioaerosols containing microorganism pathogens for prevention and control of the outbreaks of epidemic airborne diseases.

The collection and detection of bioaerosols are two important procedures for bioaerosol monitoring. Since several good review articles have summarized bioaerosol collection [1,2,3,4,17,18] and bioaerosol detection [1,19,20,21], the follow-up and emerging studies on bioaerosol collection and detection are summarized and discussed in this review to broaden the sight of the scientists and promote the studies on bioaerosol monitoring.

## 2. Bioaerosol Collection

The collection of bioaerosols is the essential prerequisite for pathogen detection, which is also an important part of bioaerosol monitoring. The bioaerosol collection methods basically rely on the physical properties of bioaerosols, such as weight and size. Currently, available bioaerosol collection methods mainly include sedimentation, filtration, centrifugation, impaction, impingement, and microfluidic chips. All these methods are able to collect bioaerosols, but their performance is susceptible to factors, such as temperature, humidity, airflow, etc. Some newly improved studies on bioaerosol collection methods are updated and compared in this section.

### 2.1. Sedimentation

Natural sedimentation was once used for bioaerosol collection since it was first proposed by Koch in 1881 using nutrient agar plates to collect the settling bioaerosols containing bacteria due to their own gravity, followed by incubation of the bacteria and enumeration of the colonies. This sedimentation was demonstrated with low efficiency by Sui et al. [22] by first using the aerosol generator to nebulize the bacterial suspension at the concentration of 10^4^ CFU/mL for mimicking bioaerosols containing bacteria in a 125-L tank, then placing the Luria-Bertani (LB) agar plates in the tank for 20 min, and finally incubating the plate at 37 °C for 24 h to count the number of the colonies. The results showed that only 26 bacterial cells were collected by this natural sedimentation. Although the sedimentation method has shown its merits of low cost, easy operation, and little impact on microbial activity, its practical applicability is greatly limited due to (1) low collection efficiency, especially for bioaerosols with smaller size (<1 μm), (2) strong external interferences, especially for complex environments, and (3) more importantly, increasing microbial risk resulting from cultured pathogenic microorganisms.

**Figure 1:**
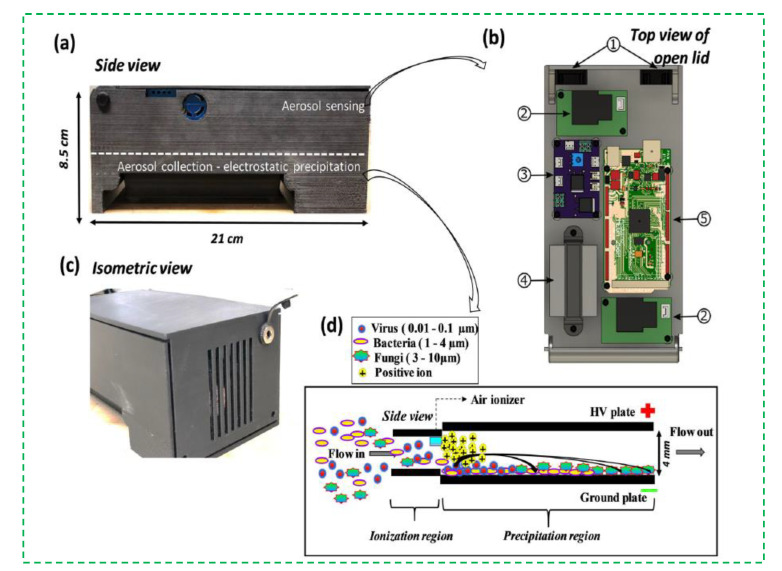
The electrostatic sedimentation for collection of bacterial bioaerosols. (**a**) side view of the device; (**b**) the aerosol sensing components; (**c**) isometric view of the device; (**d**) schematic representation of the side view of the electrostatic precipitator situated at the bottom of the device. Reprinted with permission from ref. [35]. Copyright 2021 Taylor & Francis.

Recently, sedimentation is also used with electrostatic adsorption to collect the bioaerosols [23,24,25]. An external electric field is often applied to attract or repel the charged bioaerosols due to the electrostatic effect, thus resulting in the collection of bioaerosols onto agar, liquid, and solid surfaces [26,27,28,29]. In theory, bioaerosols with more electric charges are more easily subject to electrostatic sedimentation, and vice versa. The corona discharge was reported to enable the bioaerosols to carry more electric charges, thus making the bioaerosols more easily collected onto the electrodes [30,31,32,33,34]. An interesting electrostatic sedimentation method for the collection of the bioaerosols was shown in Figure 1. A commercially available miniature air ionizer was used to generate electrical charges through corona discharge. When the bioaerosols arrived at the ionization region, they were charged by colliding with the charges and captured by an electrostatic precipitator. The results showed that the collection efficiency of the device in collecting biological particles of 1 µm was as high as 84 ± 7% [35]. The electrostatic sedimentation has shown a higher collection efficiency due to electrostatic attraction of charged bioaerosols, a better universality due to less impact from particle size, and a stronger collection capacity due to continuous-flow capture of bioaerosols. However, the activity of pathogenic microorganisms in the bioaerosols might be greatly reduced due to the electric charges, and the extremely high voltage for corona discharge is still a potential risk for practical applications.

### 2.2. Filtration

**Figure 2:**
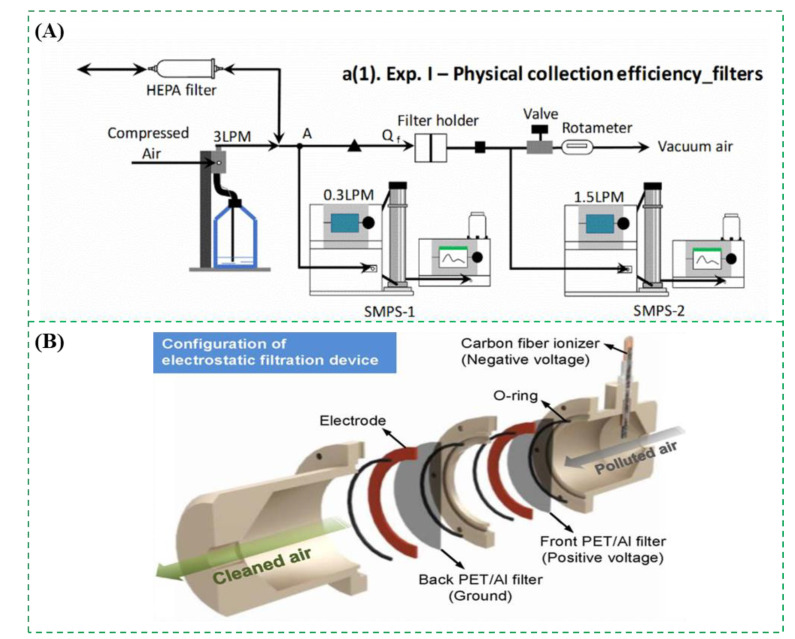
(**A**) The filtration method for collection of bioaerosols containing influenza virus. Reprinted with permission from ref. [39]. Copyright 2018 Elsevier B.V. (**B**) The filtration method for collection of bioaerosols containing *Escherichia coli* and *Staphylococcus epidermidis*. Reprinted with permission from ref. [40]. Copyright 2018 Elsevier B.V.

Filtration is frequently used to collect the bioaerosols [36,37]. The filters are often made of different materials, including polycarbonate, cellulose ester, polytetrafluoroethylene, polyvinyl chloride, nylon, gelatin, glass fiber, alumina nanofiber, and others [3,17,38]. According to the physical structure, the filters could be divided into three categories: fibrous filter, porous membrane filter, and capillary pore filter. A typical example was reported by Biswas et al. and shown in Figure 2A. A gelatin filter and a glass fiber filter were compared for physical collection of the bioaerosols containing influenza viruses. The atomizer was first used to aerosolize the viral solution to form the bioaerosols containing viruses. After they were drawn through the filter, the bioaerosols were collected on the dry film of the filter due to diffusion, interception, and impaction. Finally, the collected bioaerosols were determined using viral isolation and real-time Reverse Transcription-Polymerase chain reaction (RT-PCR). The results showed that both of the gelatin and glass fiber filters had a high collection efficiency. Compared to the glass filter, the gelatin filter had a lower stability but a higher virus recovery. However, only high concentrations of bioaerosols containing viruses could lead to positive results [39]. Another interesting example was reported by Jung et al. and shown in Figure 2B. The bioaerosols containing bacteria were first negatively charged through collision with negative ions generated by the ionizer at a constant voltage of −10 kV. When the charged bioaerosols passed through the positively charged polyester/aluminum filters, they were captured in the filters due to electrostatic interactions. The results showed that this filter had a very high collection efficiency (∼99.99%) for the bioaerosols with a geometric mean diameter of ~0.89 μm [40]. At present, many commercial filters were available, such as the button bioaerosol sampler from SKC (Covington, NC, USA) and the GSP bioaerosol sampler from BGI (Waltham, MA, USA) [2].

The filtration method has shown its merits of small size, low cost, and easy operation. However, it still has some limitations, such as (1) uncontrollable collection size due to uneven pore size of the filters, (2) inaccurate collection efficiency due to incomplete elution of the bioaerosols and easy blocking of the filters, and (3) low collection velocity due to the fragility of the filters. Besides, the complexity of the air environment and the heterogeneity of bioaerosol size also make the sampling difficult.

### 2.3. Centrifugation

**Figure 3:**
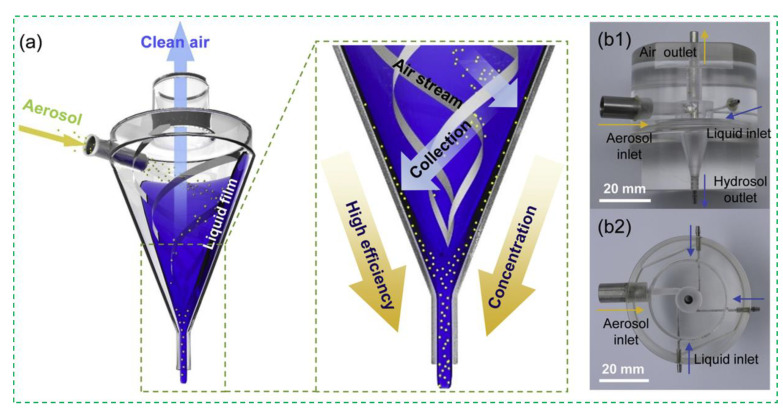
The centrifugation method for collection of the bioaerosols containing *Staphylococcus epidermidis*, *Micrococcus luteus,* and *E. coli*. (**a**) Schematic diagram of the operating principle of the wet-cyclone; (**b1**) Front- and (**b2**) top-view photographs of the wet-cyclone module. Reprinted with permission from ref. [46]. Copyright 2019 Elsevier B.V.

The centrifugation method is also reported to collect the bioaerosols. In general, the bioaerosols are injected into a special-structured chamber to form the swirling air, resulting in the centrifugation of the bioaerosols into the collection wall or liquid due to their different mass [41,42,43,44,45]. A typical example was reported by Jung et al. and shown in Figure 3. A stable thin liquid film was first formed in a conical cyclone chamber due to the centrifugation and gravity. When the bioaerosols were injected into the chamber, a helical cyclone was then formed, and the bioaerosols were finally centrifuged into the liquid film, which was drained out from the bottom, while the clean air was drained out from the top. The results showed that this centrifugation method could collect >95% of the particles with a diameter of >0.5 μm and the bioaerosols containing *Staphylococcus epidermidis* and *Micrococcus luteus* [46]. Combined with the adenosine triphosphate-bioluminescence technique, this method was able to detect the bioaerosols containing *E.coli* cells as low as 130 CFU/m^3^ [47].

The centrifugation method has shown its merits of compact size, high efficiency, and continuous-flow collection. However, it still has some limitations, such as (1) the low collection efficiency for smaller bioaerosols (<1 µm), (2) the evaporation of the thin liquid film, and (3) the difficult control of the gas and liquid flows.

### 2.4. Impaction

**Figure 4:**
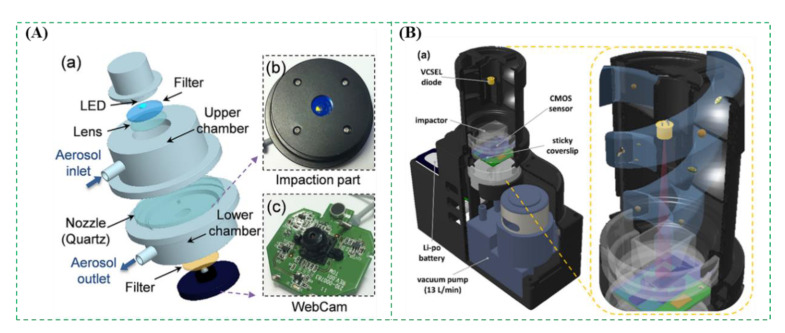
(**A**) The impactor for collection of the bioaerosols containing *Escherichia coli*. Reprinted with permission from ref. [50]. Copyright 2017 Elsevier B.V. (**B**) The impactor combined with holographic microscopy and deep-learning for detection of the bioaerosols. Reprinted with permission from ref. [51]. Copyright 2018 American Chemical Society.

The impaction method is based on inertia to collect the bioaerosols. Generally, the bioaerosols are first drawn into a nozzle using a vacuum pump. Then, the bioaerosols are impacted onto a solid collection medium (such as glass slide, agar plate, filter, gelatin, etc.), which is perpendicular to the nozzle’s outlet. Finally, the bioaerosols with higher inertia are captured onto the medium, while those with lower inertia flee with the air flow [43,48,49]. Usually, a single-stage impactor, which only needs a set of nozzles and a collection medium, has its cut-off size. The bioaerosols larger than the cut-off size can be effectively collected in the collection medium. While a multi-stage impactor is often used to study the size distribution of bioaerosols due to the gradually smaller nozzles and continuous-flow collection media. There are some commercially available impactors, such as Anderson impactors (WesTech, Salt Lake City, UT, USA), Aerotech impactors (Aerotech Laboratories, Coventry, UK), BioImpactor (AES, Combourg, France), and BioStage impactors (SKC Inc., Covington, GA, USA), etc. [2]. A typical example was shown in Figure 4A. The bioaerosols containing *Escherichia coli* cells were continuous-flow collected by the impactor onto an agarose gel with fluorescent dye and directly detected using a mini-fluorescence microscope to take in situ fluorescent images. The results showed that the collection efficiency of this impactor for the bioaerosols above a diameter of 0.84 μm was over 50% at the flow rate of 10 L/min [50]. Another example was reported by Ozcan et al. and shown in Figure 4B. An impaction method was combined with a digital holographic microscope for label-free detection and automatic classification of the bioaerosols. The bioaerosols were first collected in the substrate using the impaction method. Then, the microscope was used to record the diffraction holograms with the amplitude and phase data of each individual bioaerosol, which were further analyzed using an image processing software to classify the bioaerosols into pre-trained classes based on the deep convolutional neural networks. The results showed that this method could achieve > 94% classification accuracy for the bioaerosols [51].

At present, the impaction method has been widely applied to collect the bioaerosols because it is cost-effective, easy-to-use, and without additional post-processing. However, it also has some limitations, including (1) reduced collection efficiency due to the deposition of bioaerosols on the wall of impactors and the possible bounce of bioaerosols; (2) decreased bioactivity because of the shear force on the bioaerosols, the impaction on the collection medium and the desiccation of the bioaerosols; and (3) the difficulty of enumeration of the colonies in the collection medium due to the overlap of microorganisms.

### 2.5. Impingement

**Figure 5:**
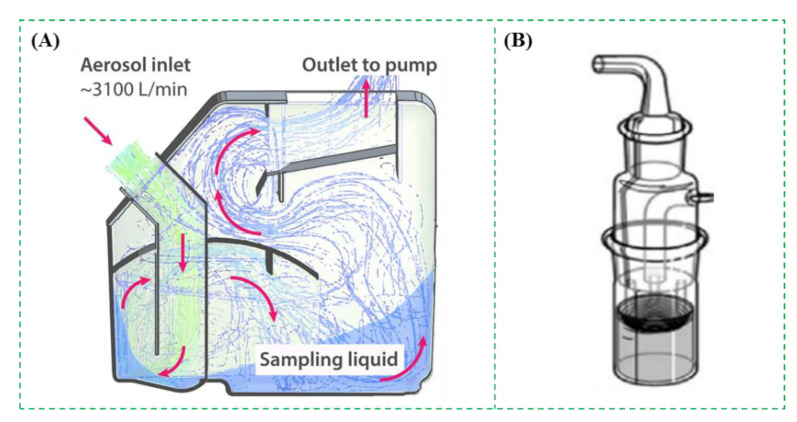
(**A**) The impinger for collection of the bioaerosols. Reprinted with permission from ref. [56]. Copyright 2017 American Chemical Society. (**B**) The liquid impinger BioSampler for collection of bioaerosol particles [57]. Reprinted with permission from ref. [57]. Copyright 2017 Elsevier B.V.

The impingement method is similar to the impaction one, and the difference is the use of a liquid collection medium. Usually, the bioaerosols are sucked into a chamber through nozzles and captured by the liquid collection medium when they strike the medium [52,53,54,55]. There are some commercial impingers, such as All-Glass Impinger (Ace Glass Inc., Vineland, USA), SKC BioSampler (SKC Inc., Covington, GA, USA), Multistage Liquid Impinger (Burkard Manufacturing Co. Ltd., Rickmansworth, UK), etc. A typical impinger was shown in Figure 5A. It was able to collect 25% of the bioaerosols with the size of <500 nm and 69–99% of those with larger sizes at the flow rate of 3.1 × 10^3^ L/min [56]. As shown in Figure 5B, the collection efficiencies of SKC BioSampler for aerosolized bacterial species from 0.5 to 10 μm were studied using an Ultraviolet Aerodynamic Particle Sizer unit. The results showed that the overall collection efficiencies of all viable biological particles were 95.3%, 87.7%, and 65.5%, respectively, for different volumes of collection liquids 20 mL, 10 mL, and 5 mL at the flow rate of 12.5 L/min [57].

These impingers can collect the bioaerosols containing pathogenic microorganisms in the liquid buffer solution, which can be directly detected without an additional elution process. Although the impingement methods have been used as a reference in many studies [58], there are some limitations for their practical applications, including: (1) reduced viability due to the shear forces in the nozzles and the turbulence caused by the air; (2) the evaporation of the liquid collection medium; and (3) the adherence of partially collected particles onto the wall of the collection chamber.

### 2.6. Microfluidics

**Figure 6:**
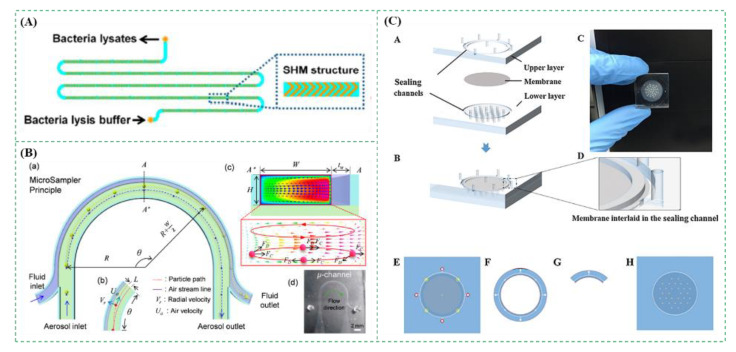
(**A**) The microfluidic chip with staggered herringbone structures for collection of the bioaerosols containing *Mycobacterium tuberculosis*. Reprinted with permission from ref. [70]. Copyright 2014 American Chemical Society. (**B**) The microfluidic chip with curved microchannel for collection of the bacterial bioaerosols. Reprinted with permission from ref. [71]. Copyright 2017 American Chemical Society. (**C**) The microfluidic chip combined with filtration for collection of bioaerosols. Reprinted with permission from ref. [72]. Copyright 2018 Elsevier B.V.

Microfluidic chips featured with miniaturization, integration, and multifunction have been widely used in environmental monitoring [59,60], food safety [61,62,63], medical diagnosis [64,65,66], etc. Recently, microfluidic chips are increasingly exploited for bioaerosol collection, which either relied on different structures of the microfluidic chips, such as the staggered herringbone for generation of the chaotic vortex [67], the curved, circle or spiral channels for production of the centrifugal forces [68], or combined with the traditional bioaerosol collection methods to develop simpler, inexpensive, and portable methods [69]. As shown in Figure 6A, a microfluidic chip with the staggered herringbone structures was designed to enrich the bioaerosols containing Mycobacterium tuberculosis. Due to the staggered herringbone structures, the chaotic flow was induced to create more chances for the collision between the bacteria and the inside wall of channels, resulting in the high capture efficiency (almost 100%) within 20 min [70]. As shown in Figure 6B, another microfluidic chip with the curved microchannel was used to collect bioaerosols into liquids. Due to the centrifugal and drag forces on the bioaerosols, the direction of bioaerosols was changed towards the liquid. The results showed that the collection efficiency was ∼90% for the bioaerosols containing *S. epidermidis* with a specific geometric mean diameter of ~0.79 μm [71]. Besides, in Figure 6C, a simple microfluidic chip combined with a microfilter achieved a high collection efficiency of 99% for bioaerosols with a diameter < 1 μm [72]. The microfluidic chips have shown their merits, such as low cost, easy integration, and automatic operation, but their low flow rate and short collection duration lead to the small sampling volume.

## 3. Pathogen Detection

The detection of pathogens is an effective method to quantify bioaerosols in the air, which is also another important part of bioaerosol monitoring. The principle, advantages, and disadvantages of pathogen detection methods are summarized in this section. Some examples with different bioaerosol detection methods were compared in Table 1. All of these methods are able to detect pathogens, but the molecular biological and immunological detection methods are obviously faster than the culture method.

### 3.1. Microbial Culture

The microbial culture is the most common method for detection of the pathogens in the bioaerosols [73,74,75,76,77,78]. Take pathogenic bacteria in the bioaerosols as an example. After the bioaerosols are collected, they are first suspended in the buffer solution and then surface plated on the agar plates, followed by incubation at the appropriate temperature for sufficient time to form the visible colonies, which are finally counted to determine the concentration of the bioaerosols. Since there are often some non-target microorganisms in the air, the non-selective culture medium can be used to cultivate all the microorganisms in the bioaerosols, while the selective culture medium can be used to cultivate only the specific microorganisms. An interesting study was reported by Schäfer et al. using the agar plates to cultivate the bioaerosols containing bacteria at the proper temperature for several days, which was further combined with subsequent colony counting procedures to analyze the diversity of the target bacteria in the bioaerosols [79,80]. The microbial culture is simple, accurate and effective; however, it has some limitations, including that it is: (1) time-consuming (over 24 h); (2) not applicable for the non-cultural pathogens; (3) not suitable for in-field applications.

### 3.2. Molecular Biological Detection

**Figure 7:**
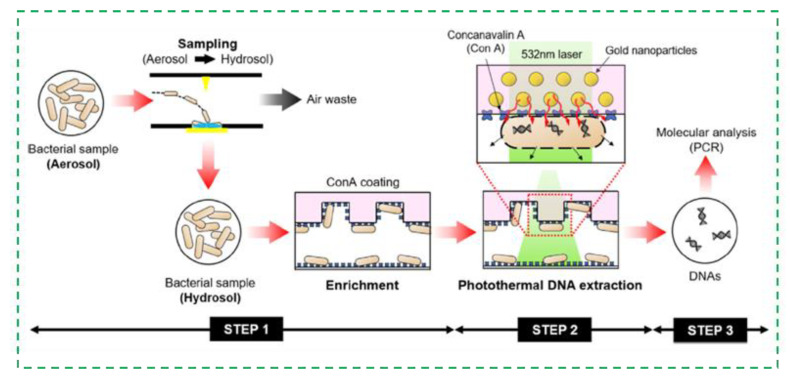
The microfluidic chip for PCR detection of the bacterial bioaerosols. Reprinted with permission from ref. [94]. Copyright 2017 Elsevier B.V.

Molecular biological detection methods have made considerable progress over the past decades [81,82]. PCR is the representative method for molecular biological detection, which is able to quantitatively and specifically detect various pathogens in the bioaerosols [83,84,85,86,87,88,89]. After the bioaerosols were collected and lysed to release the nucleic acids from target microorganisms, the nucleic acids were denatured, annealed, and extended to achieve their amplification. Hernández et al. used real-time PCR to rapidly detect *Enterococcus faecalis*, which were collected in a filter, with high sensitivity and good specificity [90]. For those bioaerosols containing RNA viruses, after RNA was extracted and reversely transcribed into DNA, the same procedure could be performed as DNA. The SARS-CoV-2 is a kind of RNA virus and could be detected using reverse transcription PCR (RT-PCR) [91]. Aoki et al. combined an IgG assay with RT-PCR to detect the SARS-CoV-2. The results showed that this combination of RT-PCR and IgG assay could improve the robustness for laboratory diagnosis of COVID-19 [92]. Recently, microfluidic chips, which are able to integrate mixing, washing, incubation, reaction and detection onto a single chip, are often used with molecular biological assays for detection of pathogens in the bioaerosols [93]. As shown in Figure 7, a microfluidic device was combined with a photothermal system to capture, lyse, and detect the bacteria in the bioaerosols. The bioaerosols were first captured by the microfluidic herringbone chip and then irradiated with a 532 nm laser, resulting in a photothermal effect of spherical gold nanoparticles in the chip and thus the thermal lysis of bacteria. Finally, the extracted biomacromolecules were analyzed for quantitative detection of bioaerosols [94]. A thermoplasmonic-assisted dual-mode transducer was presented to detect the SARS-CoV-2 viruses in 30 min, where an amplification-free-based direct viral RNA detection and an amplification-based cyclic fluorescence probe cleavage detection were combined and demonstrated as a potential tool for fast clinical infection screening and real-time environmental monitoring [95]. Besides, some other molecular biological detection methods were also applied to detect the SARS-CoV-2 viruses [96,97]. Liu et al. developed a 3D printed microfluidic chip to detect the SARS-CoV-2 viruses using a Flinders membrane for nucleic acid extraction, recombinase polymerase amplification, and loop-mediated isothermal amplification for sensitive detection, a smartphone-based platform for colorimetric signal monitoring. The result showed that this microfluidic chip was able to detect SARS-CoV-2 as low as 100 GE/mL [98].

In theory, conventional PCR is not able to discriminate viable and non-viable microorganisms, resulting in possible overestimation of pathogenic microorganisms in the bioaerosols. In recent years, some specific intercalating photo-reactive reagents were reported to pretreat the bacterial cells to form covalent bonds with the DNA of dead cells, so that only the DNA of live cells could be amplified and detected using PCR. Propidium monoazide (PMA) and ethidium monoazide (EMA) are two effective intercalating reagents, which only permeate cells with disrupted cell membrane, rendering the DNA unavailable for amplification [99,100,101]. Chen et al. developed a PMA-quantitative PCR method, which was able to quantify the viable bacteria in the bioaerosols with the linear range of 10^4^ to 10^10^ CFU/mL [102]. However, the PMA and EMA at higher concentrations are toxic, thus limiting their practical applications.

Compared to the microbial culture, the molecular biological detection methods are of rapid detection, high sensitivity, and good specificity, and have been widely applied for detection of pathogens in the bioaerosols. However, they also have some limitations, including (1) high skill due to complex nucleic acid extraction; (2) potential cross-contamination due to the leakage of nucleic acid templates; (3) laboratory dependence due to benchtop instrument.

### 3.3. Immunological Detection

The immunological detection methods are based on the antibody-antigen reaction. In the past decades, various biosensors for detection of the pathogens in the bioaerosols have received increasing attention due to their outstanding features of low cost, fast response, miniature size, and easy integration, and have been considered as a promising candidate for in-field applications in many fields, such as biomedical diagnostics, food safety, environmental monitoring, and so on [45,103,104].

**Figure 8:**
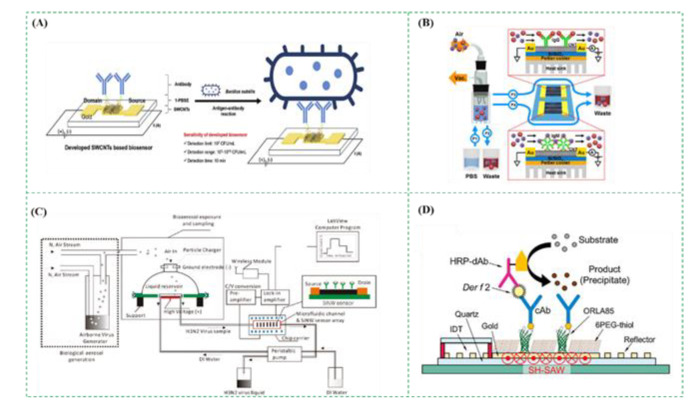
(**A**) The electrochemical biosensor for detection of the bioaerosols containing *Bacillus subtilis*. Reprinted with permission from ref. [105]. Copyright 2017 Elsevier B.V. (**B**) The FET biosensor for detection of the bioaerosols containing *Alternaria alternate* and *Aspergillus niger*. Reprinted with permission from ref. [106]. Copyright 2016 American Chemical Society. (**C**) The FET biosensor on microfluidic chips for detection of the bioaerosols containing influenza H3N2 viruses. Reprinted with permission from ref. [107]. Copyright 2011 American Chemical Society. (**D**) The SAW biosensor for detection of the bioaerosols containing house dust mite allergens. Reprinted with permission from ref. [115]. Copyright 2019 Elsevier B.V.

Electrochemical biosensors are one of the most common biosensors, which convert biological signals into electrical ones, including impedance, current, potential, etc., resulting from enzymatic catalysis, redox reaction, and antigen-antibody binding, etc. An interesting study on electrochemical biosensors was shown in Figure 8A using single-walled carbon nanotubes (SWCNTs) to detect *Bacillus subtilis* in the bioaerosols. The bioaerosols containing *Bacillus subtilis* were captured by the polyclonal antibodies immobilized on the SWCNTs-based biosensor, resulting in the change in the electric resistance, which was measured using a potentiostat. This electrochemical biosensor was able to detect *Bacillus subtilis* from 10^2^ to 1010 CFU/mL within 10 min with the detection limit of 10^2^ CFU/mL [105].

The field effect transistor (FET) biosensors are based on the measurement of drain current and have been reported to detect the pathogens in the bioaerosols [30]. The metal gates in metal-oxide-semiconductor FET structures were often replaced by ion-sensitive membranes (ISMs) and reference electrodes modified by biological elements (such as antibodies and antigens). When the charged biological targets were recognized to form the complexes on the ISMs, or the biological targets on the ISMs induced biochemical reactions to produce ionic products (such as H^+^). As a result, the density of surface charges on the ISM was changed, resulting in the change in the ISM potential and thus the change in drain current. Therefore, the drain current was measured to quantitatively determine the number of targets. As shown in Figure 8B, Lee et al. reported a two-channel carbon nanotube FET (CNT-FET) to simultaneously detect the bioaerosols containing two different fungi collected by the impinger. When the negatively charged fungi were conjugated with the antibodies immobilized on the CNT-FET, a negative gating effect was applied to the CNT channel, resulting in an increase in the current. This CNT-FET biosensor was able to simultaneously detect *Alternaria alternate* and *Aspergillus niger* from 10^1^ to 10^6^ pg/mL [106]. Besides, as shown in Figure 8C, a real-time monitoring system for the bioaerosols containing influenza H3N2 viruses was designed by integrating a FET sensor, a microfluidic chip, and a bioaerosol-to-hydrosol air sampler. When the bioaerosols containing H3N2 viruses were collected into the buffer solution by the air sampler and injected into the microfluidic chip, the viruses were captured by the antibody-modified FET sensor, resulting in the discrete conductance changes. The results showed that when the concentration of viruses increased 10 times, the FET sensor response increased about 20–30% [107].

Piezoelectric biosensors are based on the change of the mass and have been used in the fields of analytical chemistry, environmental monitoring, and gas detection. Quartz crystal microbalance (QCM) biosensors and surface acoustic wave (SAW) biosensors are two main kinds of piezoelectric biosensors. The QCM biosensor measures the change in the frequency of quartz crystal resonator resulting from the mass change. Generally, the antibodies are modified onto the gold surface of the quartz crystal resonator to specifically capture the targets in the bioaerosols, resulting in the increase in the mass on the surface and thus the decrease in the resonation frequency [108,109]. Skladal et al. proposed a QCM biosensor, which was able to detect the bioaerosols containing *Escherichia coli* with a lower detection limit of 1.45 × 10^4^ CFU/L in 16 min [110]. The SAW biosensor mainly consists of a piezoelectric material, an interdigital transducer, and an oscillation circuit, and measures the change in the viscoelasticity, mass, or frequency of a liquid [111,112,113,114]. As shown in Figure 8D, Mitsubayashi et al. developed an enhanced SAW biosensor for sensitive detection of the bioaerosols containing the house dust mite (HDM) allergens. First, the surface of the SAW transducer was modified with a self-assembled monolayer of the mixture of ORLA85, 6PEG-thiol and capture antibodies. Then, the HDM allergens, *Dermatophagoides farinae*, were dropped and incubated for 10 min. After washing with PBS, the HRP modified detection antibodies were dropped and incubated for 10 min to label the targets. Finally, the substrate was added and catalyzed by HRP for 2 min to produce the precipitates on the surface of SAW biosensor, resulting in the change in the velocity of the surface acoustic wave and thus a phase shift. The results showed that the precipitates greatly increased the mass on the surface of the SAW biosensor, and this SAW biosensor had a detection limit as low as 35 pg/mL [115].

Surface plasmon resonance (SPR) biosensors rely on the change of refractive index resulted from the mass accumulation at the sensing surface to quantify the targets from small molecules to the whole microbes, such as proteins, viruses, bacteria, and so on [116]. Similarly, the biological components were immobilized on the surface of the SPR sensor, followed by reaction with the targets, leading to the change of the mass on the surface [117]. Agranovski et al. reported an SPR biosensor for rapid detection of the bioaerosols containing bacteria. The SPR sensor was first modified with the biotinylated rabbit polyclonal anti-*E. coli* antibodies. Then, the *E. coli* cells were specifically captured by the antibodies onto the surface. The change of the refractive index was finally measured to determine the concentration of the target bacteria. This SPR biosensor could detect the bioaerosols containing *E. coli* from 1.5 × 10^3^ to 1.5 × 10^8^ CFU/mL [118]. Wang et al. developed a succinimidyl-ester-functionalized plasmonic biosensor for accurate and fast detection of total bioaerosols in different environments, which could be a reliable candidate for air quality assessment. The detection limits of this biosensor for bioaerosols containing *Escherichia coli* and *Bacillus subtilis* were 0.5119 and 1.69 cells/mL, respectively [119].

### 3.4. Others

There are also some other methods for detection of the pathogens in bioaerosols, such as flow cytometry [120], laser induced fluorescence [121,122], laser induced breakdown spectroscopy [123], epifluorescence microscopy [124,125], matrix-assisted laser desorption/ionization time of flight (MALDI-TOF) [126], Raman spectroscopy [127], transmission electron microscopy [128], scanning electron microscope [129], ATP-based bioluminescence assay [130], etc.

**Figure 9:**
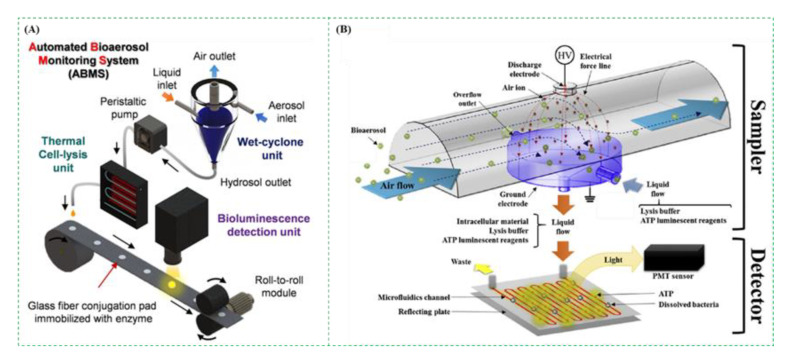
(**A**) The ATP-based bioluminescence assay for real-time detection of the bioaerosols. Reprinted with permission from ref. [47]. Copyright 2020 American Chemical Society. (**B**) The ATP-based bioluminescence assay on microfluidic chip for detection of the bacterial bioaerosols. Reprinted with permission from ref. [34]. Copyright 2016 Elsevier B.V.

Adenosine triphosphate (ATP), as an energy substance, is commonly found in live cells [131]. The ATP-based bioluminescence assay is based on the measurement of the light resulted from the reaction between luciferin and luciferase. Based on the known relationship between the light intensity and the ATP concentration, the concentration of target microorganisms in the bioaerosols could be obtained. As an available and affordable method, the ATP-based bioluminescence assay has been extensively applied for rapid detection of the pathogens in the bioaerosols [55,132,133]. As shown in Figure 9A, Jung et al. used the ATP-based bioluminescence assay for real-time detection of the bioaerosols. The luciferase-luciferin mixtures were first dropped and immobilized on the glass-fiber pad. After the bioaerosols were collected using wet cyclone and lysed using thermal lysis, the lysed sample was dropped to the pad every 2 min. The bioluminescent intensity of the pad was finally measured using the photomultiplier tube. This bioluminescence assay was able to detect the bioaerosols with pathogenic microorganisms as low as ∼130 CFU/m^3^ in seconds [47]. Besides, as shown in Figure 9B, the bioaerosols containing bacteria were first charged by the negative ions and then collected in a flowing liquid containing cell lysis buffer and ATP bioluminescence reagents. After the liquid was delivered to the microfluidic chip, the collected bacteria were dissolved by the cell lysis buffer, and ATP was extracted and detected by the bioluminescence detector. The total detection time was 30 s at a liquid flow rate of 800 µL/min [34].

This assay also showed its ability to distinguish the presence of viable but non-cultivable microorganisms. However, this assay still suffered from some limitations, including (1) significant reduction in the activity of luciferase due to prolonged exposure to the liquid phase; (2) decreased detection accuracy and stability due to the presence of other chemicals; (3) low selectivity for detection of the bioaerosols containing spores due to the low level of ATP in spores.

## 4. Conclusions and Future Trends

The monitoring of bioaerosols containing pathogenic microorganisms is very important for prevention and control of airborne diseases. The collection and detection of bioaerosols are the essential procedures for bioaerosol monitoring. The existing bioaerosol collection and detection methods basically have an impact on the bioactivity of bioaerosols, which should be further investigated to better understand the risk level of the bioaerosols and even to develop the inactivation methods for bioaerosols.

For the collection of bioaerosols, the electrostatic sedimentation, the centrifugation, the impaction, and the impingement methods might damage the bioactivity of collected bioaerosols to a certain extent, which might result in the underestimation of the risk level of bioaerosols. These methods have higher collection efficiency for collecting the bioaerosols with micrometer-scale microorganisms due to their more charges or bigger mass. Besides, these methods using liquid collection medium often have higher recovery than those using solid medium because extra washing procedure is not required. Some samplers like PTFE filters, gelatin filters, and cyclones were able to collect the SARS-CoV-2 viruses which were followed by RT-PCR analysis [138]. Besides, the microfluidic chips have shown their obvious advantages over the traditional methods and they are indeed promising to develop online collection methods. However, the concentration of pathogen microorganisms in the air is usually very low, and thus the collected bioaerosols are often few and cannot be directly detected using the existing detection methods due to their limited sensitivity. Therefore, a large volume of air samples might be used to collect enough bioaerosols for downstream detection, and more efficient collection methods should be further investigated and developed.

For the detection of bioaerosols, the microbial culture has high sensitivity and accuracy but needs a long time to get final results. PCR is fast and sensitive but needs complex DNA extraction procedures. Various biosensors with low cost, fast response, miniature size, and easy integration, have received increasing attention and been considered as effective tools for detection of bioaerosols. At present, bioaerosol monitoring generally includes two separate procedures: bioaerosol collection and pathogen detection and cannot be achieved in real-time. The integration of bioaerosol collection and detection onto a single chip is still a big challenge, but it is urgently demanded for online monitoring of bioaerosols containing pathogenic microorganisms. Besides, dead and live microorganisms always coexist in the bioaerosols, and most detection methods still cannot distinguish between dead and live microorganisms, leading to overestimation of the pathogens in the bioaerosols. Thus, it is vital to develop detection methods that are able to distinguish dead and live microorganisms.

With rapid development of the Internet of Things (IOT), bioaerosol monitoring should be tightly integrated with the IOT to achieve a more efficient response to the potential risks. The detection results for bioaerosol monitoring can be combined with the collection data (including environmental temperature, environmental humidity, sampling time, sampling location, sampling person, sample ID, etc.) and detection data (including testing person, testing time, testing method, etc.), and transmitted to bioaerosol monitoring cloud platforms through wireless communication. The detection results can be dynamically analyzed using artificial intelligence and big data technologies to predict the risk level of bioaerosols and suggest the response to this risk.

## Figures and Tables

**Table 1:** Comparison of bioaerosol detection methods.

**Detection Method**	**Target**	**Detection Range**	**Detection Time**	**LOD**	**References**
Culture	*Enterococcus*	-	24 h	10^3^ CFU/mL	[76]
PCR	*Enterococcus faecalis*	1.5 × 10^3^–1.5 × 10^8^ CFU/mL	72 min	1.5 × 10^3^ CFU/mL	[90]
RT-PCR	Bovine viral diarrhea virus	5.2 × 10^0^–5.2 × 10^8^ RNA molecules	<30 min	5.2 RNA molecules	[134]
RT-PCR	Influenza virus	3.7 × 10^4^–3.7 × 10^6^ TCID_50_/mL	<50 min	3.7 × 10^4^ TCID_50_/mL	[135]
Chemiluminescence immunoassays	*Legionella*	8 × 10^3^–8 × 10^6^ cells/mL	1 h	1 × 10^3^ cells/mL	[104]
Chemiluminescence immunoassays	*Dermatophagoides farinae*	0.49–250 ng/mL	-	0.49 ng/mL	[136]
Electrochemical biosensor	*Escherichia coli* DH5a	10^3^–10^8^ CFU/mL	20 min	150 CFU/mL	[137]
Electrochemical biosensor	*Bacillus subtilis*	10^2^–10^10^ CFU/mL	10 min	10^2^ CFU/mL	[105]
FET biosensor	*Alternaria alternate*	10^1^–10^6^ pg/mL	-	10 pg/mL	[106]
QCM biosensor	Cat allergens	5.2 × 10^0^–1.6 × 10^5^ ng/L	30 min	5.2 ng/L	[108]
QCM biosensor	*Escherichia coli*	1.45 × 10^4^–1.45 × 10^6^ CFU/L	16 min	10^4^ CFU/L	[109]
SAW biosensor	Dust mite allergens	10^0^–10^3^ ng/mL	24 min	6.1 ng/mL	[111]
SAW biosensor	Dust mite allergens	1.0–3000 ng/mL	20 min	6.3 ng/mL	[112]
SAW biosensor	Dust mite allergens	0.3–1000 ng/mL	36 min	2.5 ng/mL	[113]
SAW biosensor	Dust mite allergens	10^2^–3 × 10^3^ ng/mL	-	20.1 ng/mL	[114]
SAW biosensor	Dust mite allergens	0.08–1 ng/mL	-	35 pg/mL	[115]
SPR biosensor	MS2 phage	2.2 × 10^6^–2.2 × 10^11^ PFU/mL	<1 min	1.12 × 10^6^ PFU/mL	[116]
SPR biosensor	*Escherichia coli*	1.5 × 10^3^–1.5 × 10^8^ CFU/mL	-	1.5 × 10^3^ CFU/mL	[118]
ATP-based bioluminescence assay	*Escherichia coli*	10^3^–10^8^ CFU/mL	5 min	2.32 × 10^3^ CFU/mL	[55]
ATP-based bioluminescence assay	*Escherichia coli*	3.7 × 10^1^–3.7 × 10^7^ CFU/mL	5 min	375 CFU/mL	[47]

## Data Availability

Not applicable.
